# Supplementary material for: SPrUCE: Utilizing Ultraconserved Elements of DNA for Population‐Level Genetic Diversity Estimation
Source: Mol Ecol Resour. 2026 Apr 23;26(3):e70145. doi: 10.1111/1755-0998.70145 (PMC13106921; doi:10.1111/1755-0998.70145)
Supplement: Supplementary file 1 — Table S1: Sample metadata from Zhao et al. 2023 for Sillago sinica populations in China (Dongying, Qingdao and Wenzhou), including ID for this study, species, population, SRR ID, MEGAHIT genome size after assembly, and number of loci recovered with 400‐ and 750‐bp flanking regions. Raw data come from project PRJNA936440. Table S2: Sample metadata from Lamichhaney et al. (2015) for finch species, including ID for this study, species, population, NCBI ID, genome size after assembly, and number of loci recovered with 400‐ and 750‐bp flanking regions. Raw data comes from project PRJNA263122. Table S3: Sample metadata from Wang et al. (2024) for Apis cerana individuals from Niupeng and Zhongshui populations, including ID for this study, species, population, NCBI ID, genome size after assembly, and number of loci recovered with 400‐ and 750‐bp flanking regions. Raw data comes from project PRJNA1054499. Table S4: Sample metadata from Shi et al. (2023) for Ovis aries individuals from Oula and Panou populations, including ID for this study, species, population, NCBI ID, genome size after assembly, and number of loci recovered with 400‐ and 750‐bp flanking regions. Raw data comes from project PRJNA797957. Table S5: UCE loci recovered at different completeness thresholds (Incomplete, 50%, 80%, and 100%) for 400‐ and 750‐bp flanks. Table S6: Top: nucleotide diversity (π) and runtime (seconds) for UCE analyses using Stacked and Concatenated methods at 400‐ and 750‐bp flanking regions. Bottom: the π estimates from ANGSD and reference studies. Table S7: ANGSD computational time for angsd ‐saf and nucleotide diversity estimates (T p ). Figure S1: The weights of each site are proportional to 1 − 2/n + 1, where n is the number of elements present at the site. This function shows how weights grow and saturate for n > 10. Note that for n = 1, the weight is zero, as it should be, and as n→∞, weights go toward 1. Figure S2: (a) UCE smilogram for 400 and 750 flank sizes for Apis cerana (H [file MEN-26-e70145-s001.pdf]

Supplementary files for SPrUCE: Utilizing Ultraconserved Elements of DNA for Population-Level Genetic Diversity Estimation

Contents

S1 Supplementary Tables **3**

S2 Supplementary Figures **9**

S3 Supplementary text **17**

S3.1 SPrUCE Algorithm . . . 17

S3.2 Approximation of π_i by $2f_i$ . . . 18

List of Tables

S1 Metadata for *Sillago sinica* . . . 3

S2 Metadata for finch species across Galápagos and Cocos Island . . . 4

S3 Metadata for *Apis cerana* dataset . . . 5

S4 Metadata for *Ovis aries*. . . . 6

S5 Number of UCEs per completeness thresholds. . . . 6

S6 Summary of π and runtime (seconds) on all datasets. . . . 7

S7 ANGSD running time. . . . 8

List of Figures

S1 Weighting function. . . . 9

S2 Coverage distribution. . . . 9

S3 WGS data pre-processing pipeline for ANGSD and SPrUCE analysis . . . 10

S4 Gompertz versus uncorrected π . . . 11

S5 Impact of data subsampling, Concatenated mode . . . 12

S6 Completeness experiments. . . . 13

S7 Running time Comparisons . . . 14

S8 The fitted Gompertz functions . . . 15

S9 Hudson and Kaplan correction . . . 16

S1 Supplementary Tables

ID	Species	Population	NCBI ID	Genome Size	UCEs 400bp	UCEs 750bp
DY1	*Sillago sinica*	Dongying	SRR23814189	557 Mb	993	953
DY2	*Sillago sinica*	Dongying	SRR23814188	552 Mb	992	952
DY3	*Sillago sinica*	Dongying	SRR23814174	551 Mb	993	953
DY4	*Sillago sinica*	Dongying	SRR23814163	549 Mb	990	953
DY5	*Sillago sinica*	Dongying	SRR23814152	548 Mb	998	958
DY6	*Sillago sinica*	Dongying	SRR23814148	543 Mb	997	962
DY7	*Sillago sinica*	Dongying	SRR23814147	548 Mb	992	954
DY8	*Sillago sinica*	Dongying	SRR23814185	546 Mb	997	961
DY9	*Sillago sinica*	Dongying	SRR23814184	557 Mb	995	955
DY10	*Sillago sinica*	Dongying	SRR23814182	527 Mb	990	963
DY11	*Sillago sinica*	Dongying	SRR23814187	544 Mb	993	957
DY12	*Sillago sinica*	Dongying	SRR23814186	545 Mb	995	959
DY13	*Sillago sinica*	Dongying	SRR23814183	546 Mb	992	956
DY14	*Sillago sinica*	Dongying	SRR23814181	547 Mb	989	948
DY15	*Sillago sinica*	Dongying	SRR23814180	546 Mb	994	956
QD1	*Sillago sinica*	Qingdao	SRR23814179	549 Mb	987	948
QD2	*Sillago sinica*	Qingdao	SRR23814178	552 Mb	991	953
QD3	*Sillago sinica*	Qingdao	SRR23814177	555 Mb	990	950
QD4	*Sillago sinica*	Qingdao	SRR23814176	556 Mb	991	956
QD5	*Sillago sinica*	Qingdao	SRR23814175	549 Mb	994	956
QD6	*Sillago sinica*	Qingdao	SRR23814173	550 Mb	993	956
QD7	*Sillago sinica*	Qingdao	SRR23814172	544 Mb	994	957
QD8	*Sillago sinica*	Qingdao	SRR23814171	550 Mb	985	948
QD9	*Sillago sinica*	Qingdao	SRR23814170	554 Mb	991	953
QD10	*Sillago sinica*	Qingdao	SRR23814169	544 Mb	993	957
QD11	*Sillago sinica*	Qingdao	SRR23814168	542 Mb	987	947
QD12	*Sillago sinica*	Qingdao	SRR23814167	538 Mb	990	951
QD13	*Sillago sinica*	Qingdao	SRR23814166	544 Mb	991	952
WZ1	*Sillago sinica*	Wenzhou	SRR23814165	550 Mb	987	950
WZ2	*Sillago sinica*	Wenzhou	SRR23814164	552 Mb	996	957
WZ3	*Sillago sinica*	Wenzhou	SRR23814162	557 Mb	983	942
WZ4	*Sillago sinica*	Wenzhou	SRR23814161	551 Mb	982	943
WZ5	*Sillago sinica*	Wenzhou	SRR23814160	553 Mb	991	954
WZ6	*Sillago sinica*	Wenzhou	SRR23814159	552 Mb	986	947
WZ7	*Sillago sinica*	Wenzhou	SRR23814158	553 Mb	987	946
WZ8	*Sillago sinica*	Wenzhou	SRR23814157	547 Mb	990	949
WZ9	*Sillago sinica*	Wenzhou	SRR23814156	558 Mb	992	953
WZ10	*Sillago sinica*	Wenzhou	SRR23814155	553 Mb	989	949
WZ11	*Sillago sinica*	Wenzhou	SRR23814154	544 Mb	992	954
WZ12	*Sillago sinica*	Wenzhou	SRR23814153	550 Mb	993	956
WZ13	*Sillago sinica*	Wenzhou	SRR23814151	550 Mb	997	956
WZ14	*Sillago sinica*	Wenzhou	SRR23814150	549 Mb	981	945
WZ15	*Sillago sinica*	Wenzhou	SRR23814149	547 Mb	996	958

Table S1: Sample metadata from Zhao et al. 2023 for *Sillago sinica* populations in China (Dongying, Qingdao, and Wenzhou), including ID for this study, species, population, SRR ID, MEGAHIT genome size after assembly, and number of loci recovered with 400 bp and 750 bp flanking regions. Raw data come from project PRJNA936440.

ID	Species	Population	NCBI ID	Genome Size	UCEs 400bp	UCEs 750bp
cfE1	*Certhidea fusca*	Espanola	SRR1607346	908 Mb	3890	3884
cfE2	*Certhidea fusca*	Espanola	SRR1607350	948 Mb	4265	4257
cfE3	*Certhidea fusca*	Espanola	SRR1607351	882 Mb	4062	4049
cfE4	*Certhidea fusca*	Espanola	SRR1607354	914 Mb	3990	3979
cfE5	*Certhidea fusca*	Espanola	SRR1607356	878 Mb	3919	3906
cfE6	*Certhidea fusca*	Espanola	SRR1607357	940 Mb	4405	4390
cfE7	*Certhidea fusca*	Espanola	SRR1607359	999 Mb	4536	4525
cfE8	*Certhidea fusca*	Espanola	SRR1607361	952 Mb	4276	4271
cfE9	*Certhidea fusca*	Espanola	SRR1607363	923 Mb	4102	4093
cfE10	*Certhidea fusca*	Espanola	SRR1607348	921 Mb	4042	4031
cfC1	*Certhidea fusca*	Cristobal	SRR1607326	952 Mb	4297	4288
cfC2	*Certhidea fusca*	Cristobal	SRR1607330	911 Mb	3960	3951
cfC3	*Certhidea fusca*	Cristobal	SRR1607332	923 Mb	4187	4179
cfC4	*Certhidea fusca*	Cristobal	SRR1607334	912 Mb	4168	4095
cfC5	*Certhidea fusca*	Cristobal	SRR1607336	957 Mb	4311	4302
cfC6	*Certhidea fusca*	Cristobal	SRR1607337	959 Mb	4278	4272
cfC8	*Certhidea fusca*	Cristobal	SRR1607342	864 Mb	3881	3873
cfC9	*Certhidea fusca*	Cristobal	SRR1607343	830 Mb	3671	3661
cfC10	*Certhidea fusca*	Cristobal	SRR1607327	979 Mb	4478	4467
gcE1	*Geospiza conirostris*	Espanola	SRR1607296	922 Mb	4106	4095
gcE2	*Geospiza conirostris*	Espanola	SRR1607302	969 Mb	4417	4407
gcE3	*Geospiza conirostris*	Espanola	SRR1607306	1014 Mb	4619	4604
gcE4	*Geospiza conirostris*	Espanola	SRR1607310	960 Mb	4394	4382
gcE5	*Geospiza conirostris*	Espanola	SRR1607312	977 Mb	4447	4436
gcE6	*Geospiza conirostris*	Espanola	SRR1607316	991 Mb	4543	4531
gcE7	*Geospiza conirostris*	Espanola	SRR1607318	931 Mb	4203	4191
gcE8	*Geospiza conirostris*	Espanola	SRR1607320	991 Mb	4513	4500
gcE9	*Geospiza conirostris*	Espanola	SRR1607323	995 Mb	4571	4561
gcE10	*Geospiza conirostris*	Espanola	SRR1607299	938 Mb	4240	4229
gcG1	*Geospiza conirostris*	Genovesa	SRR1607365	962 Mb	4347	4343
gcG2	*Geospiza conirostris*	Genovesa	SRR1607369	968 Mb	4376	4364
gcG3	*Geospiza conirostris*	Genovesa	SRR1607372	881 Mb	3981	3977
gcG4	*Geospiza conirostris*	Genovesa	SRR1607373	992 Mb	4579	4563
gcG5	*Geospiza conirostris*	Genovesa	SRR1607375	913 Mb	4037	4027
gcG6	*Geospiza conirostris*	Genovesa	SRR1607377	873 Mb	4019	4009
gcG7	*Geospiza conirostris*	Genovesa	SRR1607380	878 Mb	3755	3753
gcG8	*Geospiza conirostris*	Genovesa	SRR1607382	888 Mb	3876	3872
gcG9	*Geospiza conirostris*	Genovesa	SRR1607384	898 Mb	3899	3895
pnC1	*Pinaroloxias inornata*	Cocos Island	SRR1607513	764 Mb	3399	3395
pnC2	*Pinaroloxias inornata*	Cocos Island	SRR1607514	908 Mb	4031	4028
pnC3	*Pinaroloxias inornata*	Cocos Island	SRR1607517	817 Mb	3663	3660
pnC4	*Pinaroloxias inornata*	Cocos Island	SRR1607518	843 Mb	3704	3699
pnC5	*Pinaroloxias inornata*	Cocos Island	SRR1607520	842 Mb	3604	3595
pnC6	*Pinaroloxias inornata*	Cocos Island	SRR1607522	763 Mb	3259	3257
pnC7	*Pinaroloxias inornata*	Cocos Island	SRR1607528	996 Mb	4566	4554
pnC8	*Pinaroloxias inornata*	Cocos Island	SRR1607531	894 Mb	4022	4011

Table S2: Sample metadata from Lamichhaney et al. (2015) for finch species, including ID for this study, species, population, NCBI ID, genome size after assembly, and number of loci recovered with 400 bp and 750 bp flanking regions. Raw data comes from project PRJNA263122.

**ID**	**Species**	**Population**	**NCBI ID**	**Genome Size**	**UCEs 400bp**	**UCEs 750bp**
NP1	*Apis cerana*	Niupeng	SRR27281405	214 Mb	2305	2297
NP2	*Apis cerana*	Niupeng	SRR27281404	210 Mb	2343	2336
NP3	*Apis cerana*	Niupeng	SRR27281403	206 Mb	2348	2345
NP4	*Apis cerana*	Niupeng	SRR27281402	208 Mb	2366	2361
NP5	*Apis cerana*	Niupeng	SRR27281514	211 Mb	2367	2362
NP6	*Apis cerana*	Niupeng	SRR27281513	206 Mb	2333	2326
NP7	*Apis cerana*	Niupeng	SRR27281512	212 Mb	2374	2366
NP8	*Apis cerana*	Niupeng	SRR27281511	205 Mb	2338	2329
NP9	*Apis cerana*	Niupeng	SRR27281510	207 Mb	2348	2341
NP10	*Apis cerana*	Niupeng	SRR27281509	205 Mb	2309	2303
ZS1	*Apis cerana*	Zhongshui	SRR27281517	202 Mb	2334	2328
ZS2	*Apis cerana*	Zhongshui	SRR27281516	203 Mb	2349	2341
ZS3	*Apis cerana*	Zhongshui	SRR27281489	203 Mb	2326	2319
ZS4	*Apis cerana*	Zhongshui	SRR27281446	205 Mb	2368	2360
ZS5	*Apis cerana*	Zhongshui	SRR27281435	196 Mb	2332	2322
ZS6	*Apis cerana*	Zhongshui	SRR27281424	206 Mb	2352	2345
ZS7	*Apis cerana*	Zhongshui	SRR27281477	208 Mb	2361	2353
ZS8	*Apis cerana*	Zhongshui	SRR27281466	201 Mb	2316	2306
ZS9	*Apis cerana*	Zhongshui	SRR27281455	190 Mb	2306	2296
ZS10	*Apis cerana*	Zhongshui	SRR27281412	194 Mb	2360	2353

Table S3: Sample metadata from Wang et al. (2024) for *Apis cerana* individuals from Niupeng and Zhongshui populations, including ID for this study, species, population, NCBI ID, genome size after assembly, and number of loci recovered with 400 bp and 750 bp flanking regions. Raw data comes from project PRJNA1054499

ID	Species	Population	NCBI ID	Genome Size	UCEs 400bp	UCEs 750bp
OL-1	*Ovis aries*	Oula	SRR17643011	2.75 G	4070	4050
OL-2	*Ovis aries*	Oula	SRR17643010	2.66 G	4077	4057
OL-3	*Ovis aries*	Oula	SRR17642999	2.64 G	4045	4024
OL-4	*Ovis aries*	Oula	SRR17642998	2.67 G	4026	4016
OL-5	*Ovis aries*	Oula	SRR17642997	2.60 G	4005	3986
OL-6	*Ovis aries*	Oula	SRR17642996	2.62 G	4050	4033
OL-7	*Ovis aries*	Oula	SRR17642995	2.65 G	4090	4066
OL-8	*Ovis aries*	Oula	SRR17642994	2.67 G	4106	4085
OL-9	*Ovis aries*	Oula	SRR17642993	2.54 G	4112	4094
OL-10	*Ovis aries*	Oula	SRR17642992	2.59 G	4117	4096
PO-1	*Ovis aries*	Panou	SRR17643009	2.62 G	4115	4094
PO-2	*Ovis aries*	Panou	SRR17643008	2.63 G	4076	4057
PO-3	*Ovis aries*	Panou	SRR17643007	2.64 G	4062	4045
PO-4	*Ovis aries*	Panou	SRR17643006	2.58 G	4103	4082
PO-5	*Ovis aries*	Panou	SRR17643005	2.57 G	4008	3989
PO-6	*Ovis aries*	Panou	SRR17643004	2.63 G	4034	4022
PO-7	*Ovis aries*	Panou	SRR17643003	2.67 G	4040	4018
PO-8	*Ovis aries*	Panou	SRR17643002	2.70 G	4082	4057
PO-9	*Ovis aries*	Panou	SRR17643001	2.70 G	3983	3959
PO-10	*Ovis aries*	Panou	SRR17643000	2.70 G	4031	4009

Table S4: Sample metadata from Shi et al. (2023) for *Ovis aries* individuals from Oula and Panou populations, including ID for this study, species, population, NCBI ID, genome size after assembly, and number of loci recovered with 400 bp and 750 bp flanking regions. Raw data comes from project PRJNA797957

Species Group	Population	Incomplete<br>400 / 750	50% Complete<br>400 / 750	80% Complete<br>400 / 750	100% Complete<br>400 / 750
A. cerana	Niupeng	2412 / 2409	2389 / 2382	2349 / 2340	1973 / 1965
	Zhongshui	2410 / 2402	2382 / 2373	2336 / 2326	1997 / 1987
C. fusca	Cristobal	4737 / 4729	4684 / 4674	4068 / 4053	1713 / 1706
	Espanola	4751 / 4744	4651 / 4635	3993 / 3477	1601 / 1597
G. conirostris	Espanola	4739 / 4728	4678 / 4662	4433 / 4417	2665 / 2653
	Genovesa	4739 / 4733	4655 / 4654	3933 / 3969	1694 / 1688
P. inornata	Cocos Island	4657 / 4654	4485 / 4476	3639 / 3625	1030 / 1029
O. aries	Oula	4167 / 4149	4148 / 4126	4096 / 4075	3496 / 3423
	Panou	4162 / 4143	4147 / 4125	4076 / 4051	3334 / 3314
S. sinica	Dongying	1005 / 971	1000 / 964	993 / 952	919 / 878
	Qingdao	1005 / 970	998 / 961	992 / 951	909 / 870
	Wenzhou	1005 / 970	999 / 958	988 / 947	898 / 861

Table S5: UCE loci recovered at different completeness thresholds (Incomplete, 50%, 80%, and 100%) for 400 bp and 750 bp flanks.

Species Group	Population	STACKED		CONCATENATED	
		400bp / Time(s)	750bp / Time(s)	400 bp/Time(s)	750bp / Time(s)
A. cerana	Niupeng	0.00493 / 4.7	0.00426 / 6.41	0.00481 / 15649.53	0.00424 / 27289.39
	Zhongshui	0.00504 / 3.22	0.00443 / 4.21	0.00488 / 16193.87	0.00441 / 22950.23
S. sinica	Dongying	0.00621 / 1.40	0.00589 / 3.22	0.00611 / 1725.15	0.00580 / 5093.64
	Qingdao	0.00674 / 2.62	0.00602 / 4.62	0.00653 / 5481.33	0.00594 / 5560.10
	Wenzhou	0.00711 / 3.95	0.00628 / 1.43	0.00689 / 3240.16	0.00617 / 10370.19
C. fusca	Cristobal	0.00152 / 2.17	0.00146 / 1.63	0.00154 / 15244.47	0.00151 / 28394.42
	Espanola	0.00056 / 2.62	0.00054 / 2.22	0.00052 / 5794.33	0.00053 / 8125.45
G. conirostris	Espanola	0.00195 / 2.13	0.00184 / 3.63	0.00193 / 14779.95	0.00182 / 48939.41
	Genovesa	0.00225 / 2.67	0.00225 / 2.98	0.00227 / 21278.82	0.00229 / 34843.64
P. inornata	Cocos	0.00121 / 3.03	0.00122 / 2.22	0.00121 / 9816.39	0.00119 / 14800.04
O. aries	Oula	0.00288 / 3.25	0.00279 / 3.16	0.00275 / 16673.69	0.00277 / 24245.68
	Panou	0.00265 / 2.51	0.00270 / 5.21	0.00258 / 25657.63	0.00266 / 21798.80

Species	Pop.	ANGSD π	Ref. Paper π	Reference
A. cerana	Niupeng	0.00544	0.00157	Wang et al. 2024
	Zhongshui	0.00559	0.00158	Wang et al. 2024
S. sinica	Dongying	0.00634	0.00241	Zhao et al. 2023
	Qingdao	0.00639	0.00241	Zhao et al. 2023
	Wenzhou	0.00681	0.00244	Zhao et al. 2023
C. fusca	Cristobal	0.00190	0.0013	Lamichhaney et al. 2015
	Espanola	0.00062	0.0003	Lamichhaney et al. 2015
G. conirostris	Espanola	0.00236	0.0015	Lamichhaney et al. 2015
	Genovesa	0.00277	0.0018	Lamichhaney et al. 2015
P. inornata	Cocos	0.00123	0.0007	Lamichhaney et al. 2015
O. aries	Oula	0.0039	0.00275	Shit et al. 2023
	Panou	0.00357	0.00265	Shit et al. 2023

Table S6: Top: Nucleotide diversity (π) and runtime (seconds) for UCE analyses using Stacked and Concatenated methods at 400 bp and 750 bp flanking regions. Bottom: The π estimates from ANGSD and reference studies.

**Species**	**Common name**	**Population**	**CPU time (s)**	**Walltime (s)**	T_p
Apis cerana	Honeybee	Niupeng	8837.23	55974	0.00544
Apis cerana	Honeybee	Zhonshui	8535.11	79778	0.00560
Sillago sinica	Smelt	Dongying	48188.80	201125	0.00634
Sillago sinica	Smelt	Qingdao	44783.30	185689	0.00639
Sillago sinica	Smelt	Wenzhou	52448.80	196087	0.00681
Certhidea fusca	Finch	Cristobal	30629.70	454237	0.00190
Certhidea fusca	Finch	Española	27450.97	380453	0.00063
Geospiza conirostris	Finch	Española	37672.08	261712	0.00237
Geospiza conirostris	Finch	Genovesa	33847.33	3057805	0.00277
Pinaroloxias inornata	Finch	Cocos	28902.48	292567	0.00123
Ovis aries	Sheep	Oula	103380.32	1047668	0.00390
Ovis aries	Sheep	Panou	82163.04	1049469	0.00357

Table S7: ANGSD computational time for `angsd -saf` and nucleotide diversity estimates (T_p).

S2 Supplementary Figures

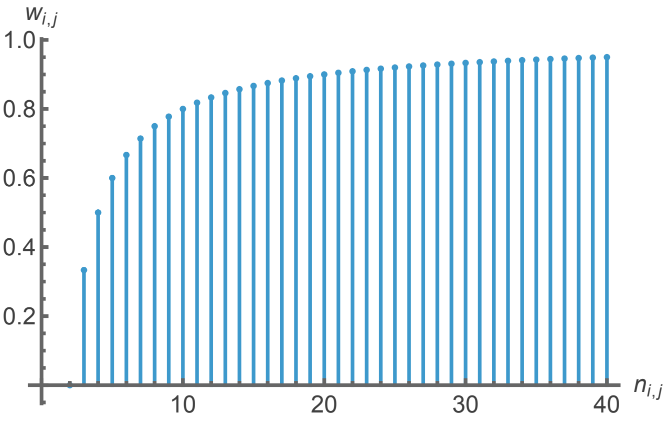

Figure S1: The weights of each site are proportional to $1 - \frac{2}{n+1}$, where n is the number of elements present at the site. This function shows how weights grow and saturate for $n > 10$. Note that for $n = 1$, the weight is zero, as it should be, and as $n \to \infty$, weights go towards 1.

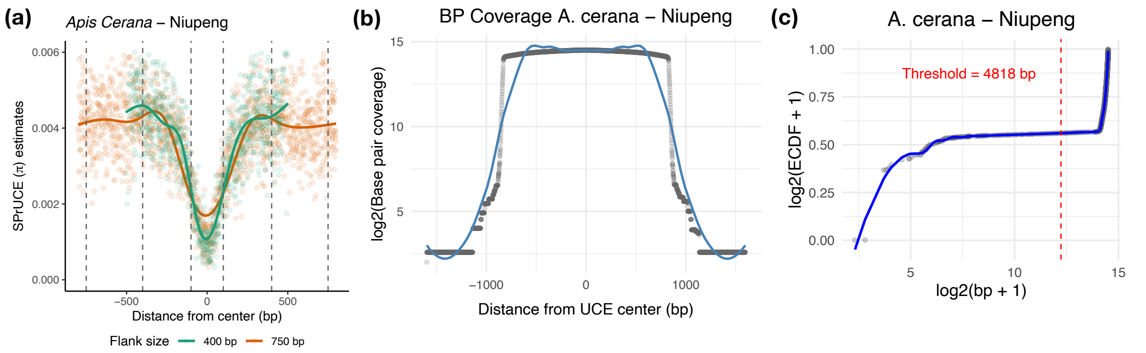

Figure S2: **(a)** UCE smilogram for 400 and 750 flank sizes for for *Apis cerana* (Honeybee – Niupeng). **(b)** Distribution of base pair coverage across positions flanking UCEs. Each point represents the log-transformed number of base pairs observed at a given position relative to the UCE center (position 0). A smoothed curve is overlaid to highlight the overall trend. Coverage is highest near the center of the UCE and decreases symmetrically toward the flanking regions. **(c)** Log-transformed spline-smoothed empirical cumulative distribution functions (ECDFs) of base pair counts. The dashed red line indicates the estimated inflection point based on the second derivative of the smoothed curve, used to define the base pair threshold for inclusion. Threshold values for bp included.

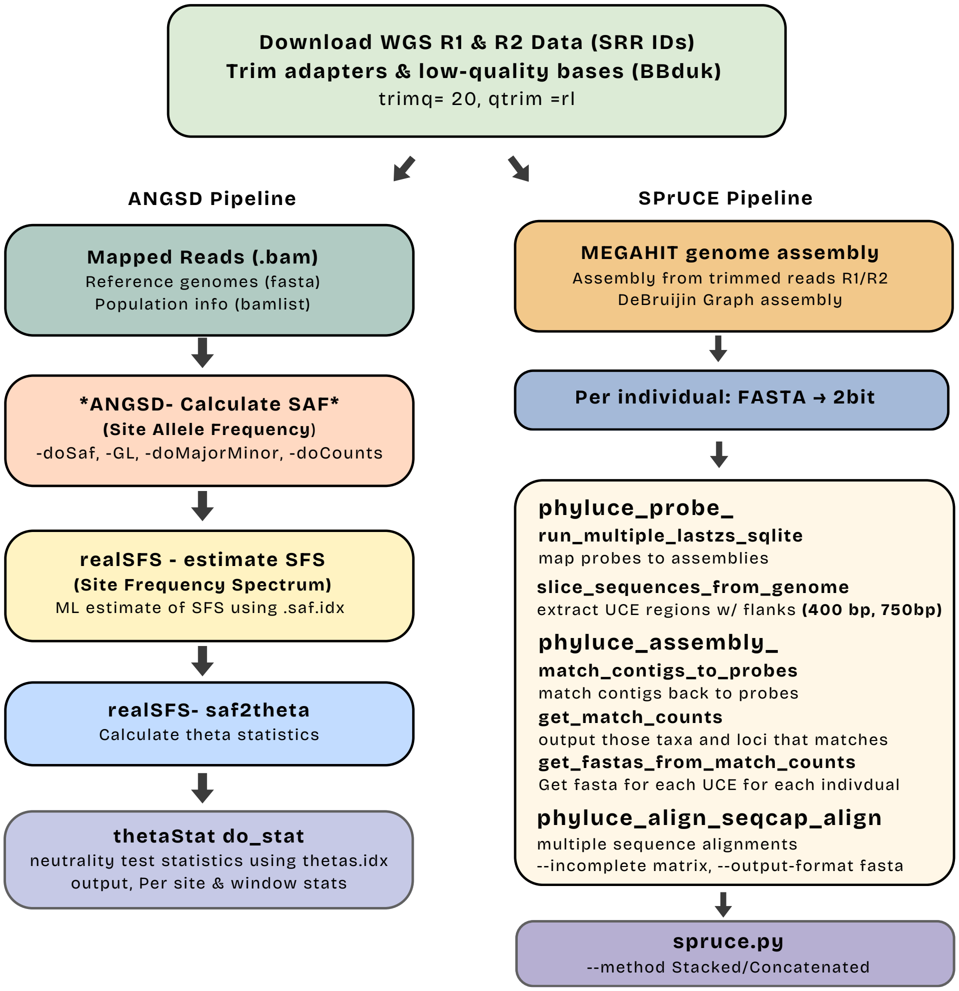

Figure S3: WGS data pre-processing pipeline for ANGSD and SPrUCE analysis

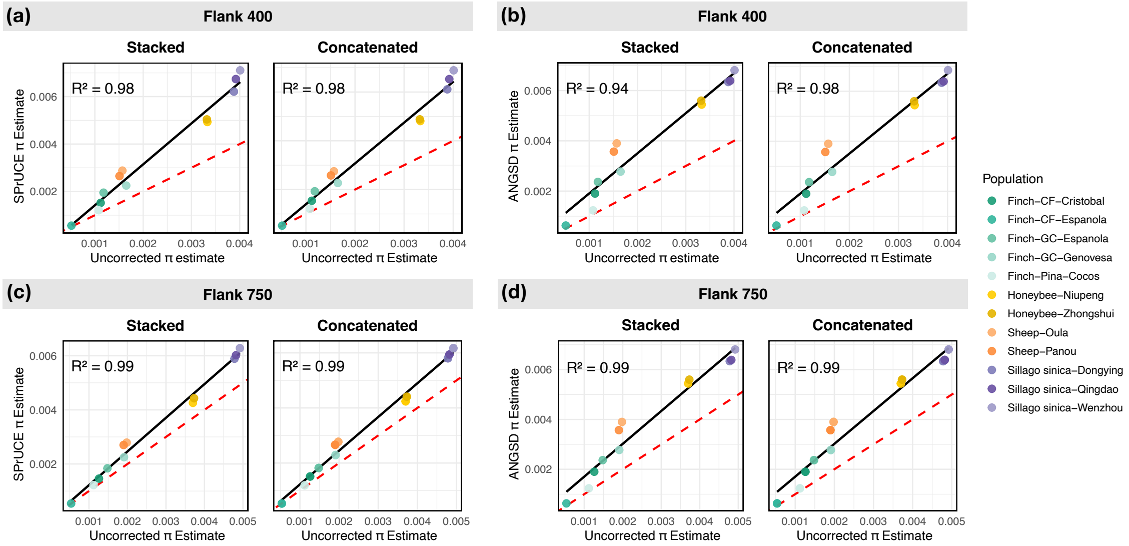

Figure S4: Comparison of uncorrected nucleotide diversity estimates with SPrUCE (left: a, c) and ANGSD (right: b, d) for 400 bp and 750 bp flanking regions. Pearson's correlation coefficient was used to assess agreement, and the coefficient of determination (R^2) is reported for each comparison. The dashed red line represents the 1:1 relationship ($x = y$), and the solid black line shows the fitted linear regression.

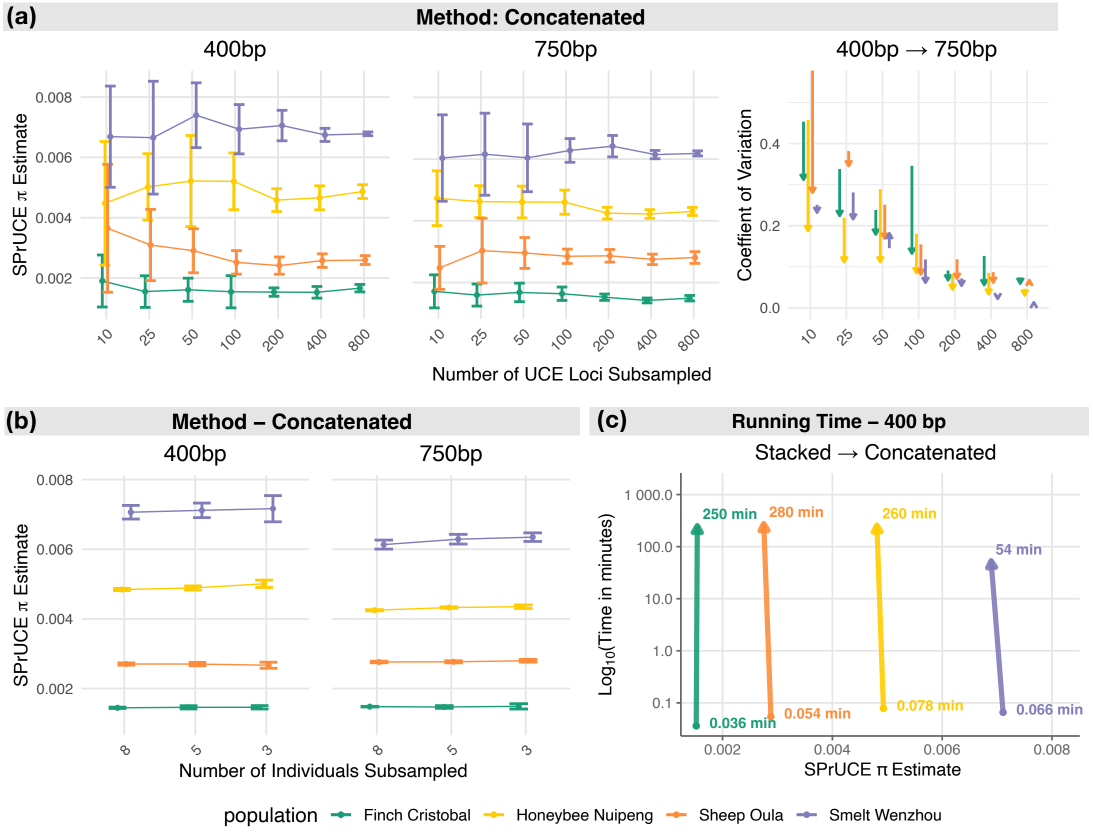

Figure S5: Estimates of nucleotide diversity π from random subsampling of UCE loci and individuals. (a) SPrUCE π estimates from subsampling UCE loci (10–800 loci, ten replicates each) using the Concatenated mode at 400 bp and 750 bp flanking regions; coefficients of variation are shown at right. (b) π estimates obtained by varying the number of individuals per population (3, 5, and 8 individuals, ten replicates each) using the Concatenated mode. (c) Runtime comparison between the Stacked and Concatenated modes at 400 bp flank length using a single CPU core. Population colors correspond to Finch (Green), Honeybee (Yellow), Sheep (Orange), and Smelt (Purple).

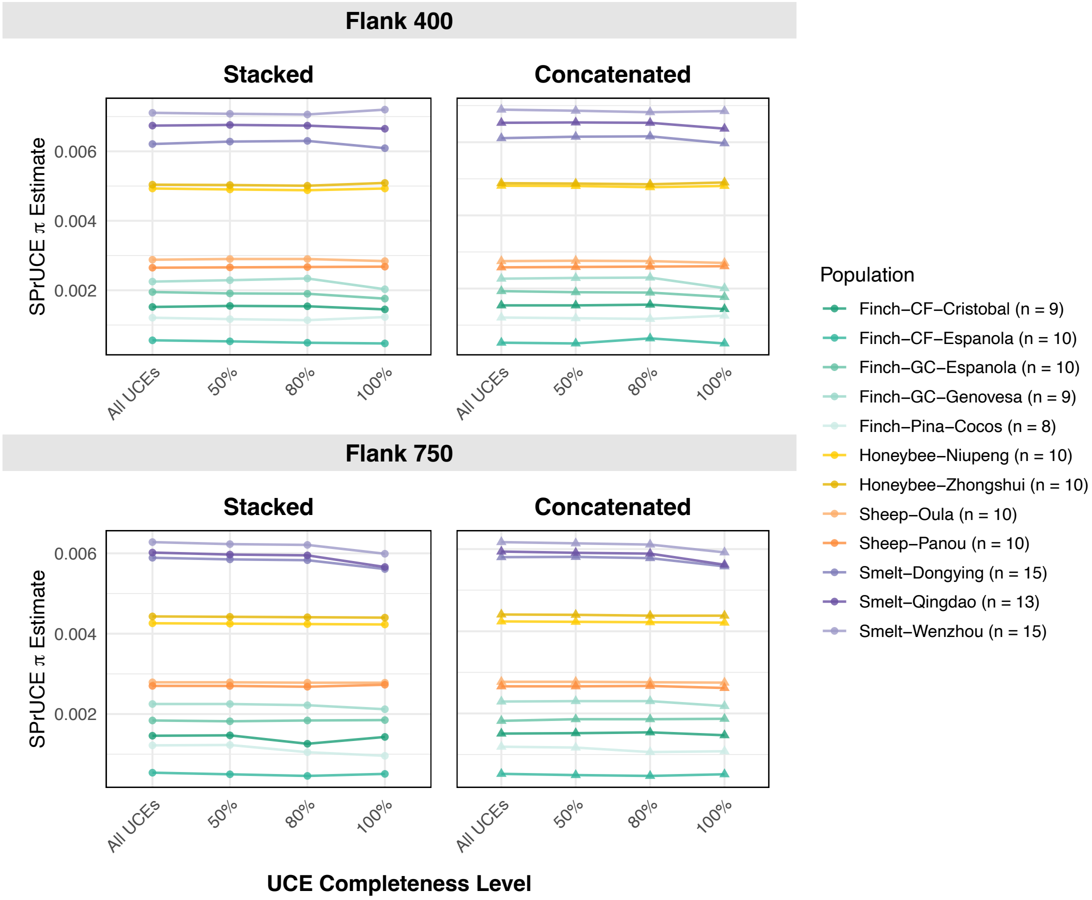

Figure S6: Estimated nucleotide diversity (π) across dataset completeness thresholds for all populations. Completeness thresholds were defined based on the minimum proportion of individuals required per UCE locus: All UCEs (no filtering), 50% (at least half of individuals present per locus), 80%, and 100% (all individuals present in every locus). The top panels show results for 400 bp flanking regions, and the bottom panels show results for 750 bp flanking regions. Each flank length includes estimates from the SPrUCE Stacked mode (left) and Concatenated mode (right). Population sample sizes (n) are shown in the legend.

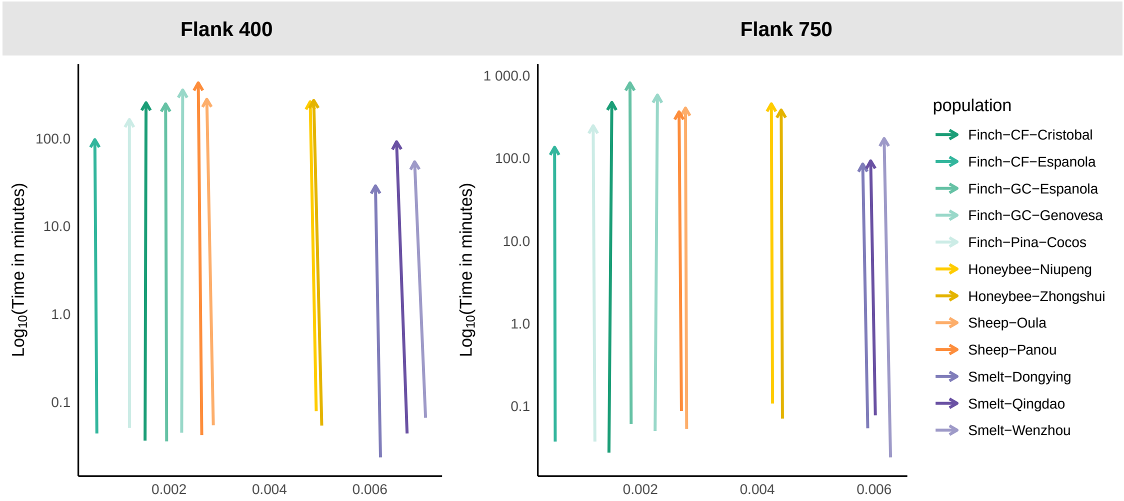

Figure S7: Comparison of runtime (y-axis) and nucleotide diversity estimates (x-axis) across methods and populations. Showing the relationship between log-transformed run time (in minutes) for Stacked to Concatenated methods. Results are grouped by flank length group: 400 bp flank and 750 bp flank.

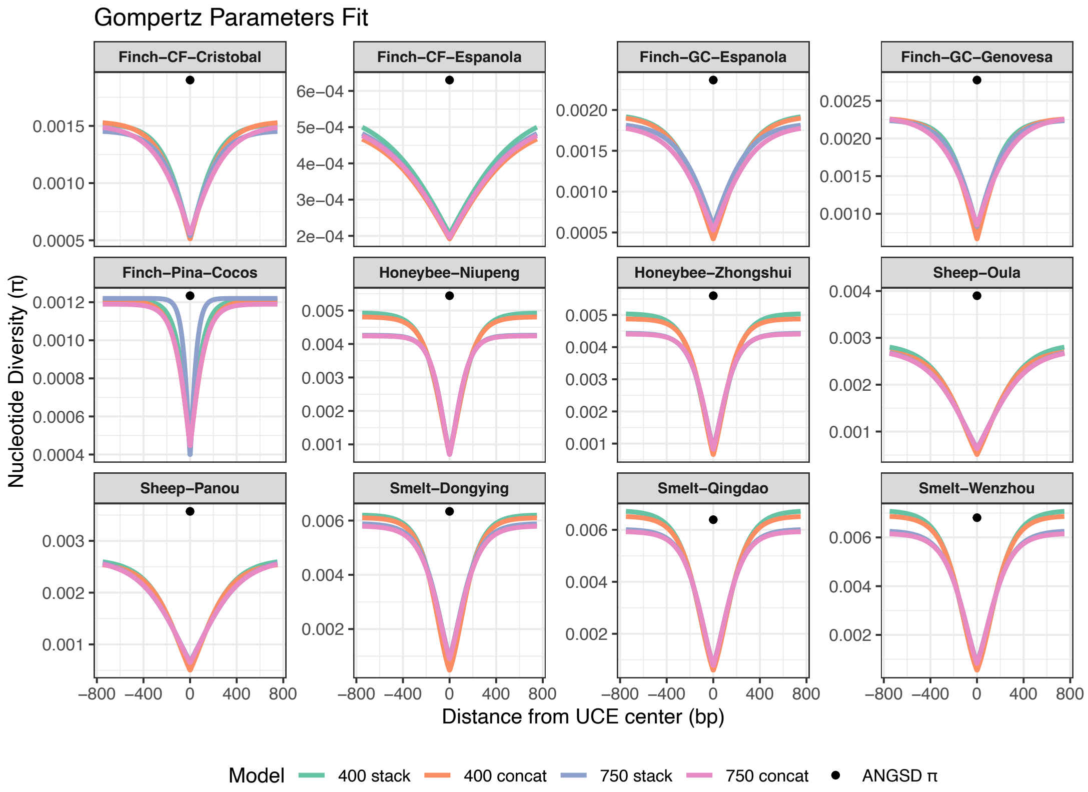

Species Group	Population	Size	Stacked 400		Concat 400		Stacked 750		Concat 750	
		n	β	γ	β	γ	β	γ	β	γ
A. cerana	Niupeng	10	1.855	0.00959	1.915	0.01032	1.806	0.01284	1.839	0.01307
	Zhongshui	10	1.943	0.00936	2.030	0.01037	1.661	0.01031	1.704	0.01067
C. fusca	Cristobal	9	1.089	0.00733	1.110	0.00658	1.004	0.00696	1.000	0.00552
	Espanola	10	1.007	0.00291	1.002	0.00298	1.011	0.00292	1.001	0.00299
G. conirostris	Espanola	10	1.492	0.00592	1.534	0.00604	1.149	0.00577	1.245	0.00520
	Genovesa	9	1.200	0.00793	1.237	0.00717	1.005	0.00675	1.000	0.00553
P. inornata	Cocos island	8	1.021	0.01449	1.000	0.01190	1.160	0.02647	1.000	0.01261
O. aries	Oula	10	1.700	0.00549	1.700	0.00593	1.478	0.00480	1.486	0.00487
	Panou	10	1.645	0.00583	1.650	0.00612	1.402	0.00447	1.420	0.00468
S. sinica	Dongying	15	2.543	0.00980	2.572	0.00995	1.852	0.00895	1.875	0.00892
	Qingdao	13	2.364	0.00837	2.418	0.00888	2.030	0.00885	2.054	0.00908
	Wenzhou	15	2.513	0.00817	2.554	0.00860	1.996	0.00803	2.029	0.00833

Figure S8: The fitted Gompertz function across species and populations. Estimates of β and γ reflect the shape and decay rates of diversity decline with distance from UCE centers for both 400 bp and 750 bp flanking regions. The parameters estimated in each case is shown in the table below. Note how low values of γ correspond to slower rates of reaching the asymptote, and coincide with higher deviations from the ANGSD estimates.

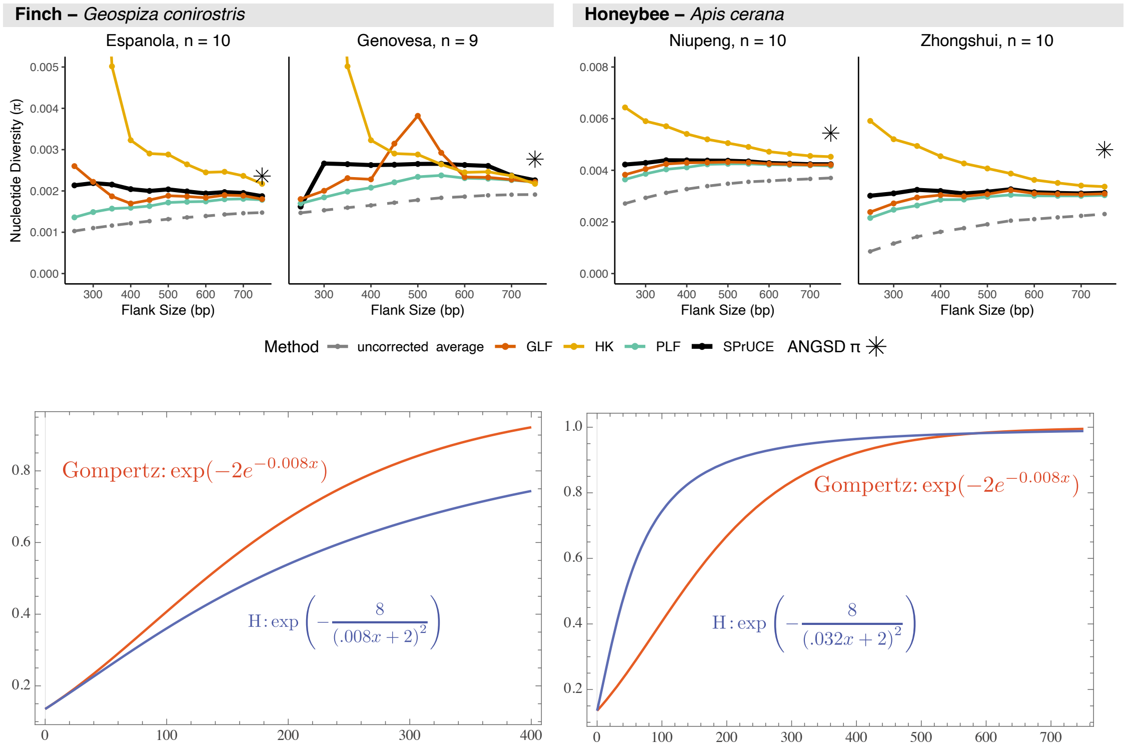

Figure S9: Top: Using the Hudson Kaplan equation ($\exp\left(-\frac{u\cdot s}{(2r\cdot x+2s)^2}\right)$) for modeling change in diversity versus other functions, including Gompertz. HK is far more sensitive to flank size; reducing the flank below 600bp can lead to very high estimates. Lack of robustness of HK discouraged us to use it, despite its theoretical appeal. Bottom: The HK equation has a slower rate of reaching its asymptote compared to Gompertz with equivalent parameters that, according to calculations, make the two functions approximate each other (left). The approximation is only good for small x. Given larger flank (right), if we chose parameters that force HK to get near the asymptote at about 750bp (by increasing the recombination rate r four-fold), the HK model will predict a faster increase in diversity close to the core compared to Gompertz. Note that HK equation is convex, while Gompertz has an inflection point.

S3 Supplementary text

S3.1 SPrUCE Algorithm

procedure EstimateDiversity
  for each UCE alignment A_j in alignments **do**
    $i \leftarrow 0$
    for each position in the alignment A_j **do**
      Identify major allele as the most frequent base (consensus)
      Categorize deviations as substitutions, insertions, or deletions
      if $|\text{non-gap alleles}| \leq 2$ **then**
        $i \leftarrow i + 1$
        $n_{i,j} \leftarrow$ Number of non-gap sequences in the column
        $s_{i,j} \leftarrow$ Number of times the major allele appears in the column
        $\pi_{i,j} \leftarrow$nucleotide diversity at position i computed using $\frac{s_{i,j} \cdot (n_{i,j} - s_{i,j})}{\binom{n_{i,j}}{2}}$
      end if
    end for
  end for
  if mode = `concat` **then**
    Estimate (θ, β, γ) by minimizing

$$\sum_{j=1}^{k} \sum_{i=-\ell}^{\ell} \frac{n_{i,j} - 1}{n_{i,j} + 1} \left(f(i; \theta, \beta, \gamma) - \pi_{i,j} \right)^2 .$$

  else ▷ mode = `stack`
    $\forall i : n_i = \sum_j n_{i,j}$
    $f \leftarrow$ A spline function fit to the log of empirical cumulative distribution of $\log(n_i)$.
    $\ell' \leftarrow$ The inflection point of f where its second derivative changes sign.
    Estimate (θ, β, γ) by minimizing

$$\sum_{i=-\ell'}^{\ell'} \frac{n_i - 1}{n_i + 1} \left(f(i; \theta, \beta, \gamma) - \frac{1}{k} \sum_{j=1}^{k} \pi_{i,j} \right)^2$$

  end if
  return The diversity estimates (θ) and other Gompertz parameters (β, γ)
end procedure

Algorithm S1: **SPrUCE.** Algorithm

S3.2 Approximation of π_i by $2f_i$

1. For a site i, let s_i be the count of individuals with the derived allele at site i, and n be the number of individuals.

2. Let f_i be the fraction of alleles that are one at position i.

3. Tajima's π is estimated using by averaging the following quantity across sites:

$$\pi_i = \frac{s_i \cdot (n - s_i)}{\binom{n}{2}} \tag{S1}$$

$$\simeq 2\frac{s_i(n - s_i)}{n \cdot n} \tag{S2}$$

$$= 2f_i(1 - f_i) \tag{S3}$$

$$= 2f_i - 2f_i^2 \tag{S4}$$

$$\approx 2f_i \tag{S5}$$

   The approximation in Equation (S2) follow from $n^2 \gg n$ for a substantially large n and the approximation in Equation (S5) follows from $f_i \gg f_i^2$ for a small value of f_i, such as 0.01 or lower.

4. The `phyluce_align_get_smilogram_from_alignments` function in Phyluce outputs f_i for each position along the UCE, stacking all positions i of all UCEs on top of one another, effectively making n to be the number of individuals multiplied by the number of loci, which is often a very large number.

5. Calculations above show that if the "substitution frequency" quantity outputted by Phyluce is to be interpreted as genetic diversity, and if we decide to ignore the impact of selection, we still need to multiply the "substitution frequency" by a factor of 2.
